# Modulating brain integrative actions as a new perspective on pharmacological approaches to neuropsychiatric diseases

**DOI:** 10.3389/fendo.2022.1038874

**Published:** 2023-01-09

**Authors:** Manuela Marcoli, Luigi F. Agnati, Rafael Franco, Pietro Cortelli, Deanna Anderlini, Diego Guidolin, Chiara Cervetto, Guido Maura

**Affiliations:** ^1^ Department of Pharmacy, Section of Pharmacology and Toxicology, University of Genova, Genova, Italy; ^2^ Interuniversity Center for the Promotion of the 3Rs Principles in Teaching and Research (Centro 3R), Pisa, Italy; ^3^ Center of Excellence for Biomedical Research, University of Genova, Genova, Italy; ^4^ Department of Biomedical, Metabolic Sciences and Neuroscience, University of Modena and Reggio Emilia, Modena, Italy; ^5^ CiberNed Network Center for Neurodegenerative diseases, National Spanish Health Institute Carlos III, Madrid, Spain; ^6^ Molecular Neurobiology laboratory, Department of Biochemistry and Molecular Biomedicine. Universitat de Barcelona, Barcelona, Spain; ^7^ School of Chemistry, Universitat de Barcelona, Barcelona, Spain; ^8^ Department of Biomedical and NeuroMotor Sciences (DIBINEM), Alma Mater Studiorum, University of Bologna, Bologna, Italy; ^9^ Istituto di Ricovero e Cura a Carattere Scientifico (IRCCS) Istituto delle Scienze Neurologiche di Bologna, Bologna, Italy; ^10^ Centre for Sensorimotor Performance, The University of Queensland, Brisbane, QLD, Australia; ^11^ Department of Neuroscience, University of Padova, Padova, Italy

**Keywords:** allosteric modulators, brain connectomics, drug targets, G-protein coupled receptors (GPCRs), neuropsychiatric disorders, penta-partite synapse, receptor heteromerization, receptor mosaics

## Abstract

A critical aspect of drug development in the therapy of neuropsychiatric diseases is the “Target Problem”, that is, the selection of a proper target after not simply the etiopathological classification but rather the detection of the supposed structural and/or functional alterations in the brain networks. There are novel ways of approaching the development of drugs capable of overcoming or at least reducing the deficits without triggering deleterious side effects. For this purpose, a model of brain network organization is needed, and the main aspects of its integrative actions must also be established. Thus, to this aim we here propose an updated model of the brain as a hyper-network in which i) the penta-partite synapses are suggested as key nodes of the brain hyper-network and ii) interacting cell surface receptors appear as both decoders of signals arriving to the network and targets of central nervous system diseases. The integrative actions of the brain networks follow the “Russian Doll organization” including the micro (i.e., synaptic) and nano (i.e., molecular) levels. In this scenario, integrative actions result primarily from protein-protein interactions. Importantly, the macromolecular complexes arising from these interactions often have novel structural binding sites of allosteric nature. Taking G protein-coupled receptors (GPCRs) as potential targets, GPCRs heteromers offer a way to increase the selectivity of pharmacological treatments if proper allosteric drugs are designed. This assumption is founded on the possible selectivity of allosteric interventions on G protein-coupled receptors especially when organized as “Receptor Mosaics” at penta-partite synapse level.

## 1 From neuropsychiatric symptoms to the “Target Problem” or how to select a suitable target for designing an efficacious drug treatment

Let us open the article with a quote from Sir James Black (Nobel Laureate 1988):

*… the benefits that molecular biology will bring to pharmacology are likely, I believe, to be circumscribed by the state of physiological knowledge, models, and concepts (1).*

Based on the brain hyper-network (BHN), a model of brain operation previously proposed by us (2), we here present a rational approach to finding therapeutic targets by discussing data obtained at molecular level in the concept of the “Target Problem” (TP).

A crucial point is the assessment of the criteria to characterize a neuropsychiatric disease and to develop the scientific diagnostic approaches to detect the structural and/or functional alterations in the brain areas that could be the cause of the disease (3). From these preliminary steps, the TP would be how to proceed to develop the proper drug-based therapy to address symptoms and/or disease progression without triggering deleterious side effects. Thus, the basic assumption is that one or more brain structures and functions derail from the physiological standard and such disarrangement should be detected based on a model of brain operation.

**Figure 1:**
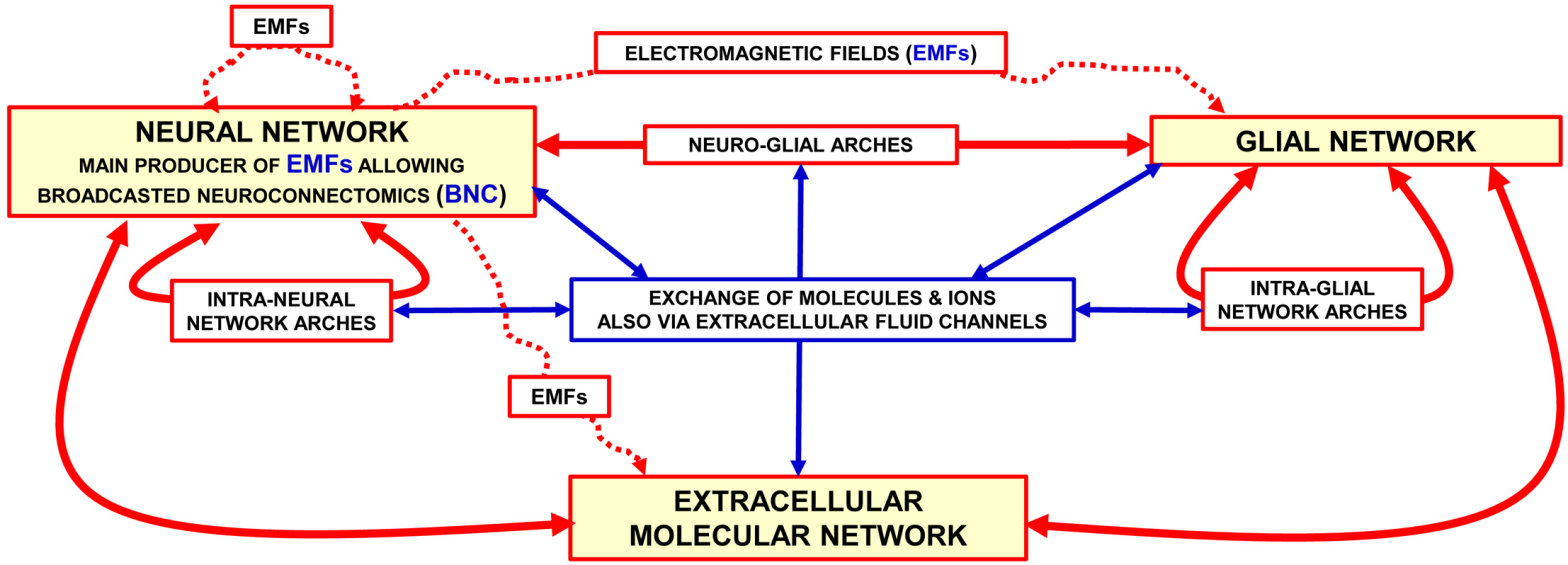
Representation of the brain as a “Brain Hyper-Network” (see the original article (2). The brain as an ″hyper-network″ formed by the integrated assemblage of neural, glia and extracellular molecular networks often organised in compartments of a different size and delimited by plastic boundaries (2). The extracellular molecular network is produced and dynamically modulated by both neurons and glial cells. In turn, the extracellular molecular network plays a role in the formation and dynamic modulation of neuro-glial, intra-neural and intra-glial arches assemblages of components of the three networks form compartments (e.g., functional modules) delimited by plastic boundaries; compartments contain circuits organised according to a ″Russian Doll pattern″ (4). This means that macro-scale, mesoscale, micro-scale, and nano-scale circuits can be described within each Functional. Module. Modified from (2). Abbreviations: EMF electromagnetic fields. For further details, see (2).

As the schematic view in **Figure 1** illustrates the BHN model for the morpho-functional organization of the central nervous system (CNS) considers the brain as made up by functional modules within plastic boundaries; these modules interact with each other by means of multiple Wiring Transmission (WT) and Volume Transmission (VT) signals (2).

According to the BHN model, several structural components at different miniaturization levels should be considered in the investigations of brain complex integrative actions. Indeed, in addition to neural networks other networks such as glial, microglial, extracellular molecular, and brain interstitial fluid (BIF) channel networks must be considered. All these networks are assembled into the BHN *via* multiple communication modes (5). In addition to the multifaceted aspects of communication modes operating at macro-scale network level, drug development has to explore the location and biochemical features at the meso-scale (cell circuit), micro-scale (cell) and nano-scale (molecular interactions) levels (6). Actually, synaptic contacts play a relevant role at the mesoscale and microscale levels, where neurotransmitter release and decoding processes take place. These highly plastic processes involve the microscale and nanoscale level, that is, cellular and molecular mechanisms occurring at the chemical synapse. Therefore, a detailed analysis of the morpho-functional organization of the CNS especially at the micro-scale and nano-scale level is of relevance to address the topic of drug development in the frame of the TP concept (7).

To clarify the main topic of the present minireview a brief historical summary of the approach that has been followed for decades for drug development focused on the micro-scale and nano-scale levels will be given. In other words, in the context of the TP some main characteristics of the drugs acting as agonists or antagonists on cell surface neuronal receptors will be summarized, with particular reference to G protein-coupled receptors (GPCRs). Subsequently, an expanded view of synaptic contacts will be presented from the perspective of microscale and nanoscale mechanisms at the penta-partite synapse (PPS).

## 2 The “Target Problem” in the context of the chemical synapse

**Figure 2:**
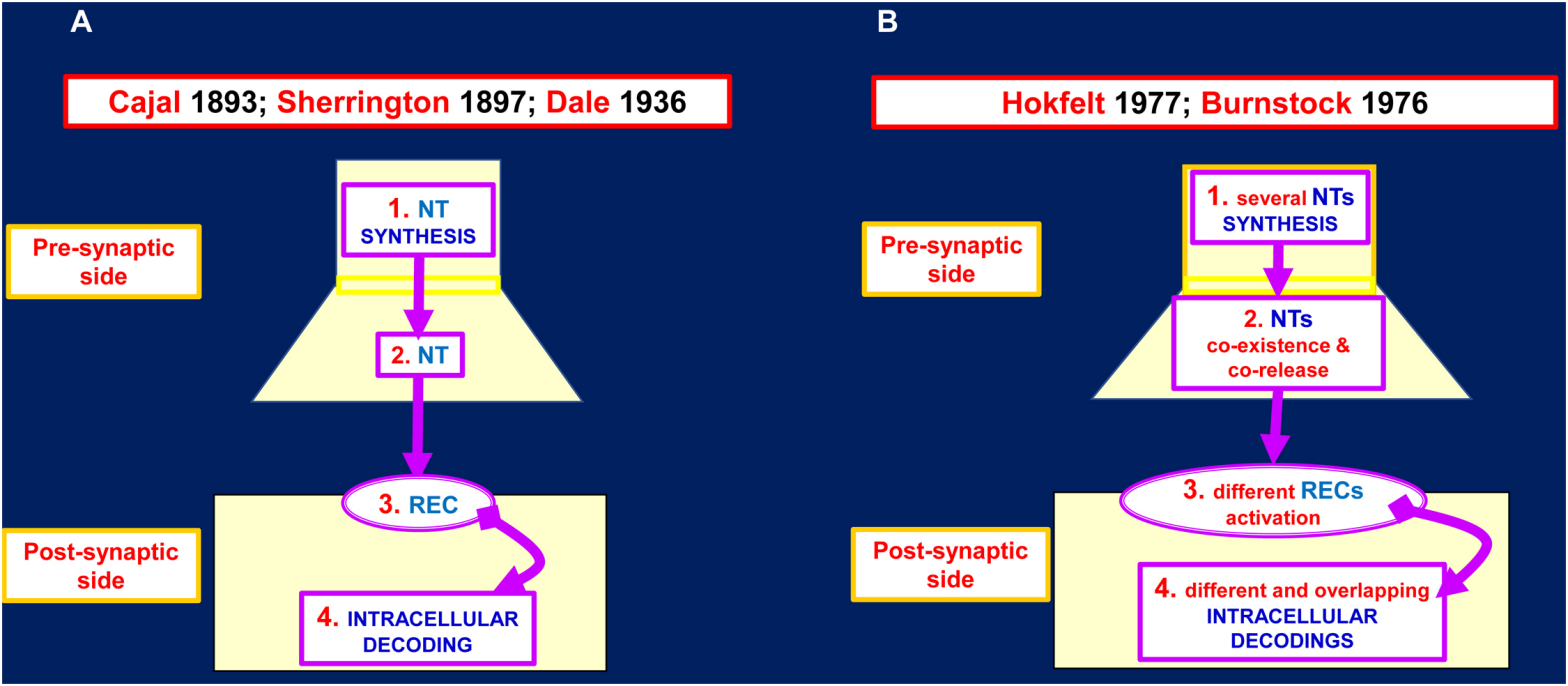
Proposed models for the chemical synapse. **(A)** Several neurotransmitter (NT) inputs like the one indicated in the scheme could impinge on the postsynaptic side. The integrated inputs result in the postsynaptic output: FIRE/NOT FIRE. In principle the pharmacological interventions could be devised on any of these four steps of the chemical synapse. **(B)** Several NT inputs like the one indicated in the scheme could impinge on the postsynaptic side. The integrated inputs result not only in the postsynaptic output: FIRE/NOT FIRE but also in complex biochemical adjustments. In principle the pharmacological interventions should be devised in a much more complex context. For further details see (8) and the herewith cited bibliography. See also (9–14). NT, neurotransmitter; REC, receptor.

In neuropsychopharmacology the classical view of synapse (see **Figure 2**, left panel)^1^ is still the most followed reference framework on which drug discovery and development are based. Indeed, early findings suggested that by acting at the synaptic receptor level, marked changes in integrative brain functions could be achieved ( (17); see (18) for a review on pioneering works discovering dopamine and neurotransmitter effects in brain; see also (19) for a discussion on effects of targeting specific receptors on brain integrative functions). However, frequent failure of drugs in drug development and/or drug side effects, especially during chronic treatments, indicated that the TP was not well resolved by this direct approach (e.g., see (20) for N-methyl-D-aspartate (NMDA) receptor as drug target). A first step forward was the characterization of iso-receptors (i.e. receptor subtypes) at synaptic level, hence the possibility of acting on a more selective target, that is, on a recognition/decoding component of synaptic transmission capable of triggering some peculiar responses at synaptic level (e.g (21). for discovery of dopamine receptor subtypes; see (22) for receptor subtypes as neurotransmitter subtle ways to modulate neuron function; see (23) for dopaminergic transmission and discussion on dopamine receptor subtypes as targets in neuropsychopharmacology; see (24) for serotonergic transmission and discussion on serotonergic receptor subtypes as targets in neuropsychopharmacology). Again, TP was not fully solved with those more selective drugs targeting iso-receptors due to treatment failure and/or side effects, which were less severe than previously (23–25).

A further important progress was the discovery by Burnstock (26, 27) and by Hokfelt (28–30) of the co-existence and co-release of different neurotransmitters from the same presynaptic terminal (**Figure 2** right panel). These breakthrough findings prompted our group to propose that at synaptic level, the co-existence and co-release of neurotransmitters should be accompanied by co-decoding of the released neurotransmitters thanks to i) the assembly of different cell surface receptors complexes (31, 32) and ii) the allosteric interactions derived from receptor-receptor interactions (RRI) (33, 34). RRIs leads to the formation of receptor dimers and multimers, also known as Receptor Mosaics (RMs) (35), the assembly of which into the native conformation may be regulated by other proteins and membrane lipids ( (36) and references therein). As pointed out in several papers, RRIs were found to occur at presynaptic, postsynaptic level and also in glial cells (for reviews see (37–41); see also (42, 43)). The available biochemical evidence on GPCR complexes and on the modulation of their structure and function by cell membrane proteins and lipids could open up new scenarios in the context of the BHN integrative actions and, accordingly, in the pharmacological approaches to neuropsychiatric diseases.

**Figure 3:**
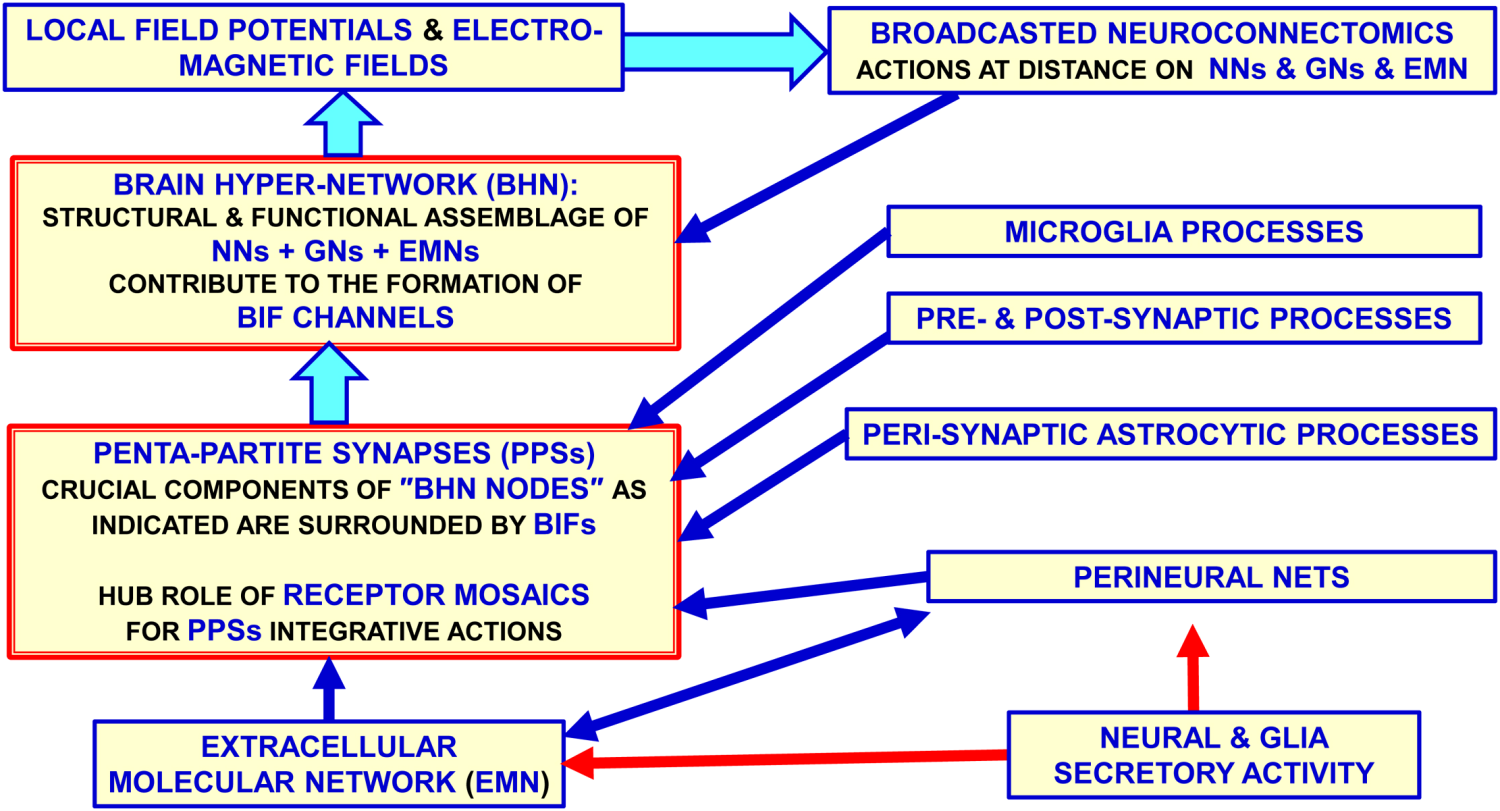
Glial cells, extracellular matrix and neurons build up penta-partite synapses (PPSs): penta-partite synapses are crucial components of the Brain Hyper-Network (BHN) nodes and are surrounded by Brain Interstitial Fluid (BIF) channels that allow a control of signals exchange between the synapse and the extra-cellular fluid. It should be noted that some highly pervasive signals as the electromagnetic field do not need the BIF channels to reach the PPS components. While neurons, astrocytes and extracellular matrix are structural components of the synapse, microglia can be mainly involved in transient functional regulating roles at the synapse in normal brain homeostasis and in disease. Modified from (2). BHN, Brain Hyper-Network; BIF, Brain Interstitial; Fluid, GNs glial networks; NNs, neural networks.

**Figure 4:**
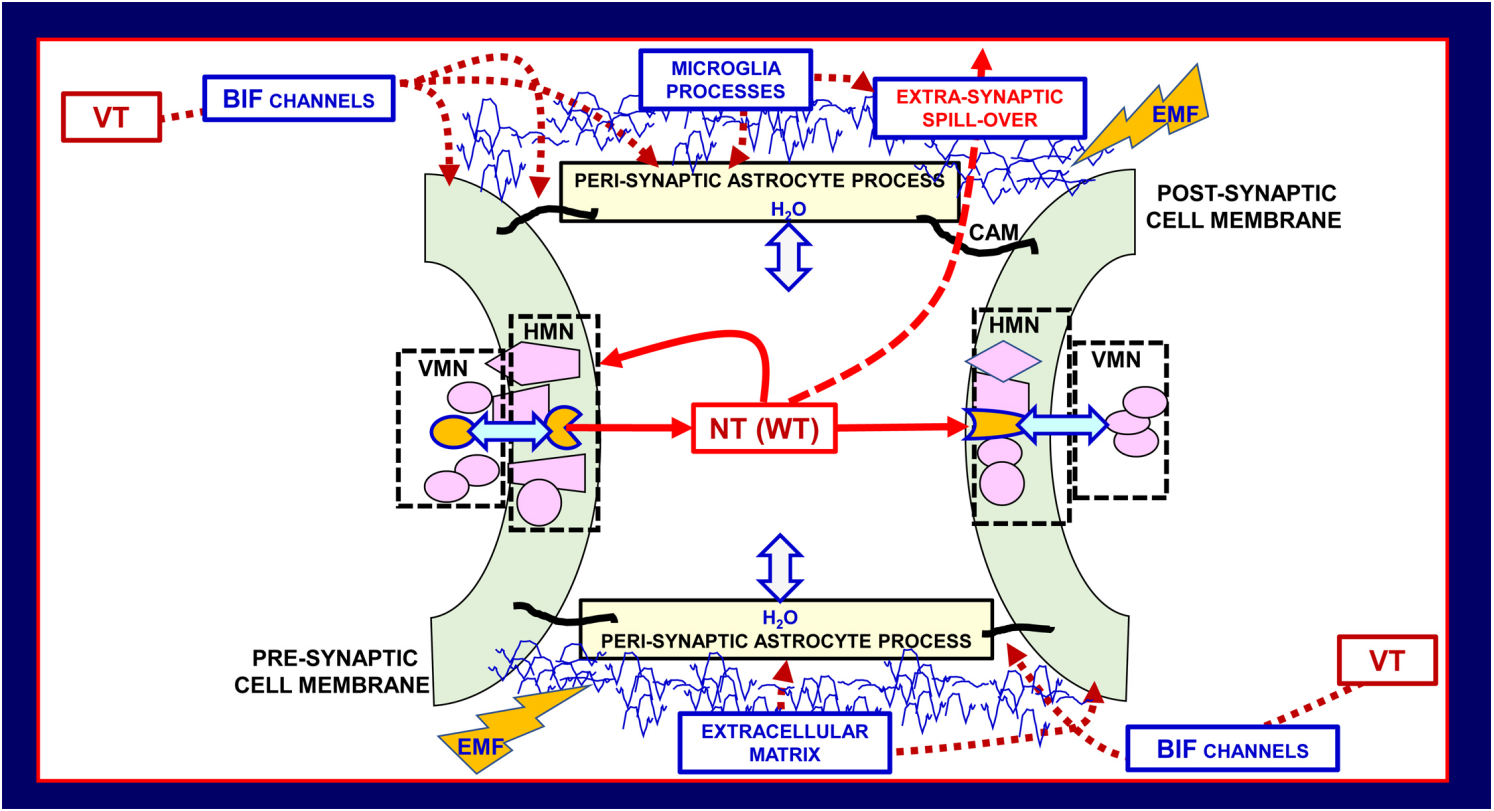
Schematic representation of the main morpho-functional features of the penta-partite synapse (PPS). Astrocytes, extracellular matrix, and neurons together with microglia build up PPS. VT signals can reach the PPSs *via* the Brain Interstitial Fluid channels (BIF channels) impinging on them. Besides the classical pre- and post-synaptic sides, of basic importance for the integrative function of the PPSs are the several molecular components indicated in the scheme, and microglial cytokines/chemokines involved in brain homeostasis and in homeostasis loss in disease. Modified from (2, 44). CAM cell adhesion molecules, EMF, electromagnetic field; HMN, horizontal molecular network; NT, neurotransmitter; VMN, vertical molecular network leading to signal transduction; VT, volume transmission; WT, wiring transmission.

BHN has as crucial nodes the PPS, which acts as complex computational structures formed by neural (pre- and post-synaptic), astrocytic, microglial and extracellular matrix molecular networks; in coordinated fashion they compute by signals spreading *via* the BIF channels (**Figures 3**, **4**). It was originally proposed the concept of tripartite synapse (45, 46), where pre- and post-synaptic structures, and the perisynaptic astrocyte processes (PAPs) ensheathing the synapse, integrate their functions. It became then apparent that a comprehensive view of the synapse includes the extracellular matrix playing not only plastic but also signal-modifying roles at the tetra-partite synapse (44, 47–50). Furthermore, the involvement of microglial cells that can transiently contact the synapse and extracellular matrix and send signals to be integrated in PPS was recognized; in particular, microglia appear to be involved in fine tuning of neural circuits in pathological conditions (51–56). Therefore, it is proposed that PPSs are likely basic computational modules playing crucial integrative tasks in healthy and in diseased conditions.

By affecting the perviousness of BIF channels controlling the signals impinging on the synapses, perisynaptic astrocytic processes regulate the multiplicity of events at the PPS. In addition, other highly pervasive signals can reach the PPSs. In this context some cues should be mentioned, namely electromagnetic fields , biophotons, oxygen and carbon dioxide, and the gaseous transmitter nitric oxide, since they can modulate morpho-functional aspects of the various networks mentioned above, deeply affecting penta-partite synapses (57–60). In particular, as far as electromagnetic fields are concerned, they are generated by neural activity and exert a direct action on the voltage sensor of the voltage-gated calcium channels that are involved in the neurotransmitter release (61–64). In this context the concept of broadcasted neuro-connectomes has been proposed to describe how these highly pervasive signals may affect information handling of brain networks especially at high miniaturization levels (2). Broadcasted neuro-connectomes, indeed, could modulate nanoscale “computational nodes” such as the RMs at the PPSs (**Figure 4**) (2, 44, 58).^2^

Membrane receptor complexes were, therefore, proposed as key integrators capable of converting multiple extracellular signals into appropriate cellular biochemical responses (37, 41, 65). These aspects, opening new possibilities to addressing the TP, will be to some extent detailed in the section that follows.^3^

## 3 Allosterism due to receptor-receptor interactions: A new player to address the “Target Problem”

Different postsynaptic decoding processes can result from the corelease of different neurotransmitters (signals) from the presynaptic terminals. Usually, chemically different transmitters are packaged in separate populations of synaptic vesicles, hence multiple signals can be released in response to a proper stimulation. However, multiple signals can be also released in the case in which two or more chemically different transmitters are present in the same vesicle (66). The regulatory mechanisms leading to co-transmitter packaging are still not completely clarified (67–70). These transmitter signals can be recognized by different decoding mechanisms both at pre- and post-synaptic level (66, 71).

These mechanisms involve molecular networks (see (72) for a review), also known as Horizontal Molecular Networks (HMNs) (**Figure 4**); they are located in membrane microdomains of presynaptic and postsynaptic elements, and in the membrane of associated glial cells. HMNs can operate as ‘‘intelligent devices” since they can sense not only extra-cellular and the intra-cellular environments but also the different components of the multi-facet structure of the penta-partite chemical synapse such as, e.g., components of the Extracellular Molecular Network (**Figures 1**, **3**).

A crucial role in HMNs may be played by GPCR complexes. A given GPCR or different GPCRs can be present in the plasma membrane as monomers, dimers or oligomers and these different molecular arrangements have important functional implications. In fact, in view of the allostery phenomenon, GPCRs at HMNs level operate as highly plastic integrative units since allosterism resulting from RRIs can be differentially modulated (2, 40, 72–75). As a matter of fact, “allostery” can be described as the biochemical phenomenon that allows the transmission, inside and between associated macromolecules, of information from the site where the effector binds to a distant functional site where the GPCR agonist binds (76). Thus, a complex regulation of the activity of macromolecules allows the appearance, *via* the macromolecular assembly, of novel properties (see (34) and the bibliography herewith mentioned). Therefore, in this context is often cited the famous Monod’s sentence stating that “allostery” for protein functions should be considered as “the second secret of life,” since it is only second as importance to the genetic code (77–79). As our group has discussed in previous papers, the actions of the orthosteric and allosteric ligands of the GPCRs that form an assembly provide the cellular decoding apparatus with sophisticated dynamics (80) in terms of binding modulation, G-protein-mediated signaling and selectivity, receptor desensitization, and switching to β-arrestin-dependent signaling (see (81) for a recent review).

In this context, the consequences of receptor complex formation deserve consideration in GPCR-based drug discovery; there are several allosteric modulators in clinical trials (82) thus showing the potential of allosteric modulators of GPCRs in multiple CNS disorders (83). Apart from decreased adverse side effects (83), allosteric ligands can provide greater receptor subtype selectivity as well as temporal selectivity (82). In this respect, novel specific allosteric sites susceptible of being allosterically targeted may appear in the quaternary structure resulting from the assembly of the receptor protomers. Selective ligands may exist for a specific receptor structure, which is only found in a heteromeric context; this possibility further expands the possibilities of modulating the decoding processes (see (84) for a review). Indeed, those allosteric binding sites are attractive targets for drug development (40, 72, 74, 85). In the absence of crystal structures of GPCR heteromers except for the extracellular domains in the heteromers of class C GPCRs (86–89) there are reliable tetrameric models reported for class A GPCR heteromers (90–95).

It is clear that to surmise protein-protein interactions , evidence of their co-localization (i.e., close proximity (<10 nm)) at cell membrane level has to be demonstrated. Hence, co-localization of two GPCRs and the interplay of their decoding processes are preliminary experimental evidence needed to assess RRI (31, 73, 96). Thus, early evidence on RRIs was indirect evidence based on a coarse co-localization of two receptors obtained by computer-assisted immunocytochemical image analyses. Biochemical approaches led to results that showed that, in membrane preparations from discrete brain regions, the ligand binding to one of the two receptors in a heteromer modulated the binding to the other receptor ( (96, 97); see (81) for a review). Such evidence was further supported by functional studies carried out *in vivo* assessing the functional relevance of the *in vitro* findings in both physiological conditions and in animal models of CNS disease (31, 98–101). In the last few decades, several biophysical techniques have been developed that allow a direct demonstration not only of the close (<10 nm) spatial proximity but also of direct interactions between receptors in neuronal and glial cell membranes. In this context experimental approaches of particular relevance are: energy transfer-based methods, bimolecular luminescence or fluorescence complementation, total internal reflection fluorescence microscopy, fluorescence correlation spectroscopy, coimmunoprecipitation, assays based on bivalent ligands and *in situ* proximity ligation assays ( (41, 102) and herewith included bibliography). We are aware that some associations of receptors as heterodimers found in heterologous systems of expression might not exist *in vivo*, and that the addressing of receptors in specific locations of the cells allows for avoiding some heteromers. Therefore, it seems of crucial importance to obtain evidence for RRIs of native GPCRs in heteromers through both physicochemical and functional approaches.

Let us briefly examine some peculiar aspects of the RRIs due to GPCRs oligomerization centered in the RMs concept in cells forming the PPS.

It is certainly possible to have receptor colocalization without receptor heteromerization and it is also possible to have heteromerization without allosteric RRIs, when subunit interaction does not cause conformational changes in other subunits of the complex (103). However, RMs especially at the PPS are likely crucial nodes for the BHN, since these plastic mosaics can also undergo a reshuffling, including addition of new proteins (“tesserae” of the mosaic via, e.g., the Roamer-Type of VT (5, 74, 104)) or alteration in their topology and order of diffusion of the allosteric signaling in the mosaic (for a discussion of the topic and the possible role of “Hub Receptors” in the RMs see below) (40, 74, 75, 105).

RRIs not only cause marked effects on the recognition/decoding processes of dimers with respect to the monomers but also it has been demonstrated that iso-receptor dimers can have a clear-cut shift in recognition/decoding characteristics with respect to the iso-receptor monomers. For example, k-δ and δ-μ opioid receptor heterodimers show a shift (with respect to the k, δ or μ monomers) in receptor affinity and in the cell response to opioid molecules (106, 107). Another remarkable finding has been the switch detected for the D_1_R-D_2_R heteromer with a change from Gs (dopamine D_1_R) and Gi/o (dopamine D_2_R) to a Gq/11 coupling (108). Thus, also simple RMs, such the dimers, display previously unsuspected properties with respect to the component monomers; this capability underlines the likely crucial role of RRIs, especially at the PPS where they are components of a “intelligent interface” communicating the extra- and intra-cellular environments. As a matter of fact, several mechanisms allow different integrative processes in RMs formed by three or more GPCRs and this fact has led us to propose the existence of an “Hub Receptor” that in some way coordinates the activation of the various components adding a new plastic capability to the mosaic (105, 109). Thus, it has been proposed to define a GPCR in a RM as a “Hub Receptor” if it has the following characteristics:

1. it interacts with multiple partners either receptors of the RM or proteins associated with the plasma membrane;
2. it can control, at least to some extent, the sequential order according to which the individual components of the mosaic are involved in the integrative action of the mosaic.

This scenario adds a new layer to brain connectivity since brain-wide connectivity can now be described at macro-scale, meso-scale, micro-scale and nano-scale levels (for the connectome concept see (110, 111)). We have been specially interested and involved in the investigations of the integrative mechanisms for reciprocal releasing and decoding processes of multiple signals at meso-scale, micro-scale and nano-scale level in particular considering the PPSs and the RMs (2, 6, 112, 113).

In this context, our aim has been to correlate the morpho-structural characteristics of the BHN computational nodes with the manner WT and VT multi-faceted communication modes allow integrative actions. As pointed out above, it is suggested that further research should be carried out to investigate the possible crucial role that allosteric interactions play in RMs located in neuronal and glial cells, especially in the PPS.

Crosstalk between neurons and glial cells occurs thanks to both WT and VT, as evidenced by, for instance, adenosine receptors-mediated signaling. As a matter of fact, adenosine is released by neurons and astrocytes allowing complex interactions among RMs containing the four adenosine receptors that may be present in both cell types (114, 115). Adenosine receptors are considered targets for several peripheral diseases, mainly cardiovascular (116, 117), but also for various neuropsychiatric diseases (118, 119). It should be underlined that although adenosine storage in synaptic vesicles and modes for release are not fully clarified (120–123), the compound can modulate neurotransmitter release/reuptake and neurotransmission itself by, among other, actions involving neuronal excitability and synaptic plasticity (124–129). In agreement with multi-faceted adenosine actions, it has been demonstrated that adenosine receptors , especially *via* RRIs, can modulate glia–neuron and glia–glia intercellular communication, with significant consequences not only on synaptic activity but also on brain network integrative functions. Thus, it is obvious the potential relevance of investigations on the allosteric modulators of RMs containing adenosine receptors localized both in neuronal and glia cell surface (41–43, 94, 130–135).

This important topic will be discussed in the next section with an emphasis on the potential implications for the design of new pharmacological approaches to combat neuropsychiatric diseases (42, 43, 74, 133, 136, 137).

## 4 A novel pharmacological approach based on targeting allosteric sites in receptor mosaics: focus on adenosine receptors

As pointed out above and by several groups (83, 129) a new approach to advance in solving the TP is to develop allosteric modulators capable of selectively favor or reduce the selected iso-receptor response to its orthosteric ligand. Thus, allosteric modulators especially of iso-receptors in RMs represent a new therapeutic approach with the added value of probably reducing side effects compared to classical receptor agonists and antagonists.

To provide therapeutic benefit, allosteric modulators can be considered based on three properties that were outlined by Korkutata et al. (129)

1. affinity modulation of the binding of the orthosteric ligand to the receptor;
2. modulation of the receptor decoding signaling triggered by the orthosteric ligand;
3. possible effects of the allosteric modulator on the receptor conformation and/or membrane location in the absence of the orthosteric ligand.

The latter property is often underestimated even though it may play an important role in the formation and location of RMs at the cell membrane level.

Allosteric modulators can have at least three different effects on the orthosteric binding sites as far as agonist/antagonist affinity and efficacy are concerned, namely can be positive, negative or neutral allosteric modulators (138). Neutral allosteric modulators have no effects on the orthosteric ligand affinity/efficacy but by occupying the allosteric binding site inhibits the activity of positive/negative allosteric modulators.

Summing up, since the integrative actions of signals by RMs are based on inter-GPCRs interactions, allosteric modulators can play important roles in at least partially solving the TP. In other words, allosteric modulators could offer a new perspective to solve the “selectivity problem”, i.e., how to hit only the proper target avoiding side effects due to targeting receptors in cells located within not altered brain areas. In view of the possible crucial role of RMs at this BHN node, the most suitable “allosteric drugs” would be those acting on GPCRs (tesserae) of the RMs especially at PPS. In this context it is important to briefly report some experimental evidence supporting the relevance of the allosteric modulations of adenosine receptors in RMs resulting in marked modulatory shifts in their integrative actions.

One example is provided by results obtained in CHO cells stably co-transfected with dopamine D_2_ and adenosine A_2A_ receptors. The interaction of these two receptors was one of the first reported for class A GPCRs (139, 140).

**Figure 5:**
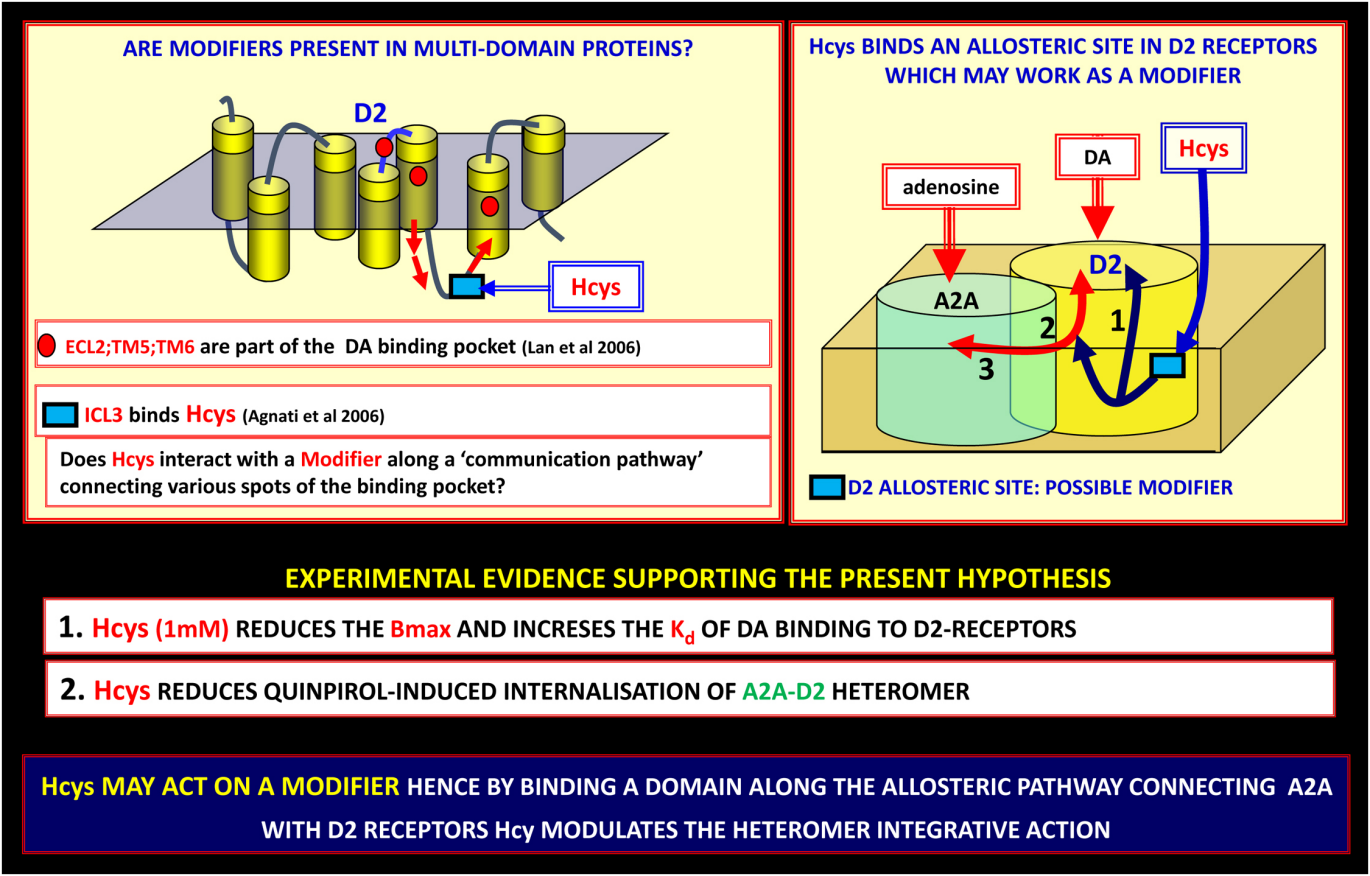
On the possible existence of modifiers along the allosteric communication channels in receptor heteromers. Allosteric communication in receptor heteromers (Receptor Mosaics, RMs) can be modulated by modifiers. Along the allosteric pathway connecting A_2A_ with D_2_ receptors, homocysteine (Hcys) can modulate the heteromer integrative action. Left panel: the Hcys allosteric binding site is an epitope on the ICL3 of D2-R, hence it can efficiently modulate two out of three of the domains involved in the binding pocket of the D_2_ receptor (for experimental procedures see (141)). This finding can be of great importance since indicates a new pharmacological approach to produce a biasing action on RMs involved in PD. Lan et al., 2006 (142); Agnati et al., 2006 (141). DA, dopamine; ECL, extracellular loop; ICL, intracellular loop; PD, Parkinson’s disease; TM, transmembrane.

In CHO cells expressing the A_2A_-D_2_ receptor heteromer, homocysteine (Hcys) selectively reduced the internalization of these heteromers induced by D2 receptor agonists stimulation. It is important to underline that Hcys did not disrupt or prevent the heteromerization of A_2A_ and D_2_ receptors, suggesting that Hcys probably behaves as a modulator of the allosteric process of energy transmission between the two partners (see **Figure 5**) (74, 105, 141). Hence, Hcys acts as an allosteric modulator that specifically binds to an *ad hoc* (allosteric) binding site made available by the structure of the heteromer.

**Figure 6:**
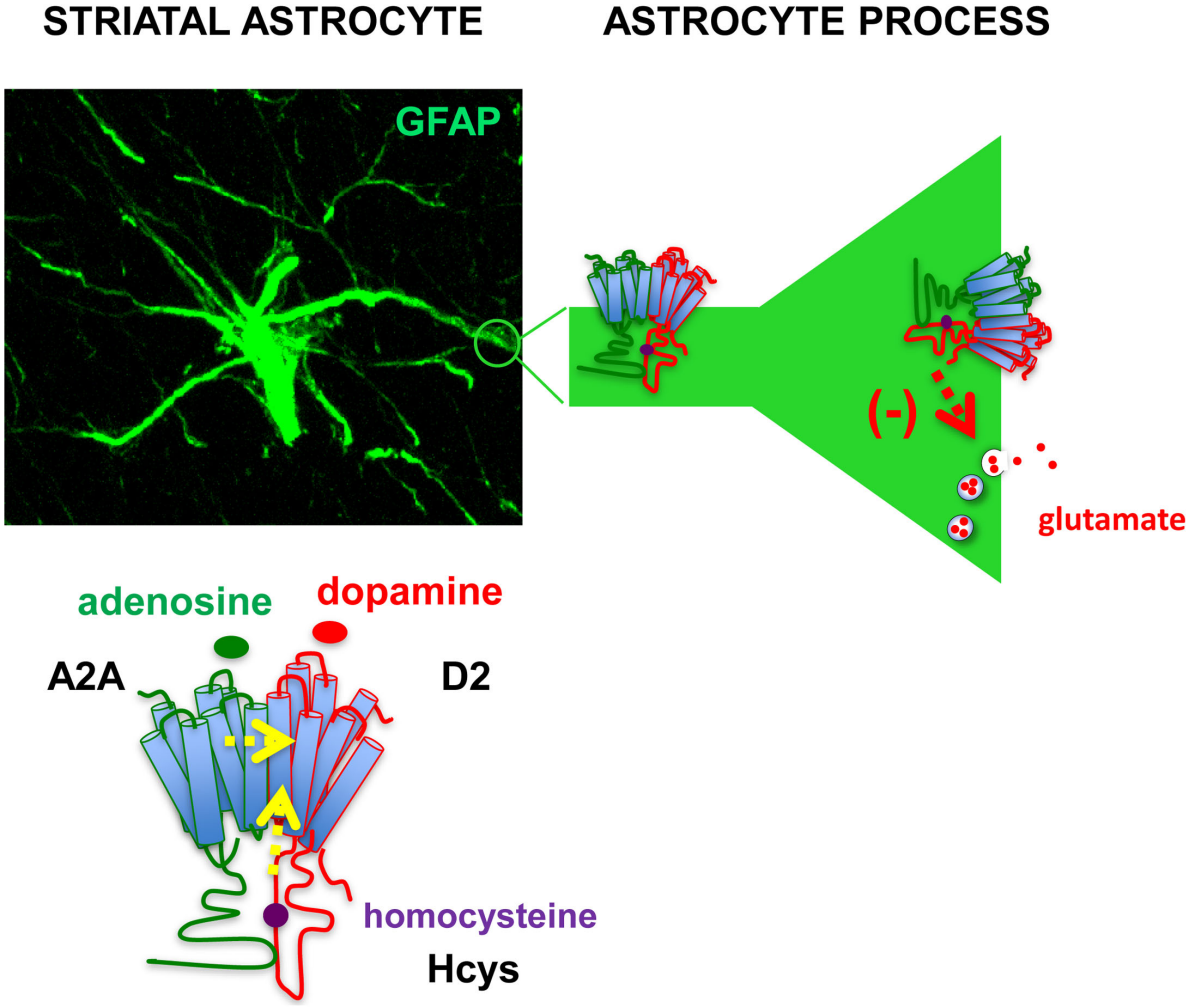
Representation of astrocytic A_2A_-D_2_ receptor heteromers in the striatum. A confocal image showing immunofluorescence staining of an astrocyte with the astrocyte marker Glial Fibrillary Acidic Protein (GFAP) in a striatal slice from adult rat. The presence of A_2A_-D_2_ receptor heteromers was demonstrated in striatal astrocytes both on perisynaptic processes and astrocyte branchlets (42, 137). Activation of the A_2A_ receptor in the heteromer prevented the effect of D_2_ receptor activation (42); intracellular homocysteine (Hcys) behaved as an allosteric antagonist of the D2 receptor while maintaining A_2A_-D_2_ interaction (136). The heteromers were involved in the control of glutamate release from the processes (42, 136). The findings suggest that A_2A_-D_2_ receptor heteromer may play crucial roles to control glutamatergic transmission in striatal functional modules, supporting exploration of strategies targeting at the heteromers to address neurodegenerative and functional striatal disorders. Yellow arrows: allosteric antagonism; red arrow: inhibition of vesicular glutamate release.

Notably, the allosteric modulation of Hcys previously reported in heterologous systems of expression was demonstrated for the associations of native receptors in heterodimers in astrocytes where Hcys reduced D_2_ receptor-mediated inhibition of glutamate release without altering the A_2A_-D_2_ receptor interaction; in fact, the A_2A_ receptor-mediated antagonism of dopaminergic actions was maintained (see **Figure 6**). These findings, as discussed also in recent published papers, open the possibility to explore novel, glia-mediated strategies to address neurodegenerative and functional BHN disorders such as Parkinson’s disease (136, 143). As a matter of fact, inhibition of astrocytic D_2_ receptor-mediated signaling by A_2A_ receptor agonists may contribute to striatal glutamatergic transmission dysfunction by increasing the extracellular glutamate levels. Thus, astrocytic A_2A_-D_2_ receptor heteromers can be a proper target to control, also by means of allosteric modulators, striatal glutamate transmission in Parkinson’s disease.

Therefore, Hcys D2 silencing at the astrocytic level could be effective targets to be considered in the context of the classical treatments aimed, inter alia, to reduce the neuro-inflammatory cascade for the Parkinson’s disease onset (74).

## 5 Concluding remarks

It is obvious that each component of the BHN can be target of a pharmacological treatment, but components that operate in an integrative mode can be more suitable targets for drug development. Proper pharmacological treatments may be those triggering synergistic effects to compensate the morpho-functional alterations of CNS diseases. For example, actions on the cell surface (e.g., Lipid Rafts) can affect RMs assemblage and composition, hence their recognition and decoding processes of signals. RMs can be modulated by, for instance, electromagnetic fields and also by the Roamer Type VT since, as mentioned above, micro-vesicles can transfer/exchange GPCRs. However, the evaluation of drug effectiveness and side effects can be made more difficult in poly-therapies involving pharmacological and non-pharmacological approaches.

According to the proposal discussed in the paper, a rational possible approach to the “Target Problem” is based on:

1. careful investigations by means of different diagnostic techniques of the BHN structures that are altered in their integrative mechanisms;
2. detection of biochemical targets (especially at the PPS level) that play a crucial role in the functional dysregulation, with special emphasis on GPCRs;
3. development of allosteric modulators that acting on the target GPCR-containing macromolecular complexes in the RMs restore the proper function of the PPS.

As far as the development of drugs acting on selected GPCRs is concerned, the development of drugs acting on dimers, hence of bivalent ligands has been also taken into account (144–153). A bivalent ligand consists of two pharmacophoric entities linked by an appropriate spacer. In this way it should be possible to target GPCR heteromers by adequate, potent, and receptor-selective pharmacophores. A potential drawback could be the hydrolysis of the compound before reaching the CNS.

Furthermore, it is worth evaluating the efficacy of drugs targeting heteromers composed of GPCRs for neurotransmitters/gliotransmitters and GPCRs for cytokines or chemokines. The combination of these different type of receptors in the RMs in the PPS, allow novel possibilities for the development of very selective allosteric modulators; this strategy might acquire relevance in the integrated signaling at PPS, i.e. when activated microglial cells are in proximity of neuronal and astrocytic synaptic structures (51, 52, 55, 56). In fact, heteromers formed by chemokine and opioid receptors in the plasma membrane of lymphocytes or cell models provide novel functional properties in response to opioids and to chemokines ( (154–156); see also (157)).

Summing up, pharmacology aimed at opposing neuropsychiatric diseases requires revisiting the target selection criteria and including the integrative modules that are morphologically and/or functionally altered in the disease. In this sense, experimental investigations should be developed that seek procedures capable of restoring, at least partially, the physiological conditions without leading to serious side effects. The development of allosteric modulators selective for structural domains in GPCR heteromers appears as a very promising strategy.

## Author contributions

All authors contributed to the article and approved the submitted version.

## Footnotes

1 It should be underlined that on this simple scheme of synaptic transmission resulting in a “firing/no-firing” of the postsynaptic neuron McCulloch and Pitts proposed a theoretical model based on Boolean logic of the CNS (15) that has been source of inspiration for decades of theoretical models of CNS integrative functions (16).

2 An interesting review on the biochemistry of RRIs and also a very valuable cultural discussion on this subject can be found in 40.

3 Knowledge of the regional distribution of a target and its presence on different CNS cell types (and on different subcellular compartment) is of pivotal importance to address the TP and to prospect a therapeutic approach to a disease, a region of the brain, or a mechanism. On the other hand, understanding the complexity of the relationships among different cell types in nodes subserving the brain integrative functions is a prerequisite to comprehend the mechanisms that can be targeted in pathological conditions. PPS where different cell types converge and signaling even from distant sources can integrate, represents the node where signaling integration leads to the system functioning, or dysfunctioning in disease. Focus on PPS may open a new mode to address the TP, through a better understanding of pathological conditions and therefore of the targetable mechanisms.
